# Bayesian salamanders: analysing the demography of an underground population of the European plethodontid *Speleomantes strinatii *with state-space modelling

**DOI:** 10.1186/1472-6785-10-4

**Published:** 2010-02-02

**Authors:** Jan Lindström, Richard Reeve, Sebastiano Salvidio

**Affiliations:** 1 Boyd Orr Centre for Population and Ecosystem Health Division of Ecology and Evolutionary Biology Faculty of Biomedical and Life Sciences University of Glasgow Glasgow G12 8QQ, UK; 2 DIPTERIS Università di Genova Corso Europa 26 I-16132 Genova, Italy and Gruppo Speleologico "A. Issel" Villa Comunale ex-Borzino, CP 21I-16012 Busalla (GE), Italy

## Abstract

**Background:**

It has been suggested that Plethodontid salamanders are excellent candidates for indicating ecosystem health. However, detailed, long-term data sets of their populations are rare, limiting our understanding of the demographic processes underlying their population fluctuations. Here we present a demographic analysis based on a 1996 - 2008 data set on an underground population of *Speleomantes strinatii *(Aellen) in NW Italy. We utilised a Bayesian state-space approach allowing us to parameterise a stage-structured Lefkovitch model. We used all the available population data from annual temporary removal experiments to provide us with the baseline data on the numbers of juveniles, subadults and adult males and females present at any given time.

**Results:**

Sampling the posterior chains of the converged state-space model gives us the likelihood distributions of the state-specific demographic rates and the associated uncertainty of these estimates. Analysing the resulting parameterised Lefkovitch matrices shows that the population growth is very close to 1, and that at population equilibrium we expect half of the individuals present to be adults of reproductive age which is what we also observe in the data. Elasticity analysis shows that adult survival is the key determinant for population growth.

**Conclusion:**

This analysis demonstrates how an understanding of population demography can be gained from structured population data even in a case where following marked individuals over their whole lifespan is not practical.

## Background

The human population relies on ecosystem services for its maintenance and well-being, and these can only be produced by functioning ecosystems [e.g. [1,2]]. It has been argued that conserving nature and thereby retaining ecosystem functions is often vastly more profitable in economic terms than converting it, for instance, to forestry or agriculture [3]. Effective ecosystem conservation requires noticing changes both initially before active management has taken place and later to assess whether an employed conservation strategy is adequate. It is not realistic to have monitoring programmes for everything so indicator species are often used to gauge the health of a larger ecosystem [4,5]. Biological properties that make a species or a taxonomic group attractive for use as indicators of ecosystem health are, for instance, clear taxonomy, existing information on basic biology, some measurable correlations to ecosystem health, limited mobility, practicality of sampling and relatively low variability of population fluctuations [5].

Once an indicator species has been identified, it is then necessary to know whether its population is stable, increasing or decreasing [6-8]. Such assessments are often based on statistical analyses of long-term population trends [9-11]. However, a much more thorough understanding of the situation can be gained by a more detailed demographic analysis whenever the extant data allow, especially when relatively small populations are considered [12-14]. This is because they are more sensititive to demographic and environmental stochasticity than larger populations and it is therefore important to understand how changes in population structure translate to changes in population growth rate [8]. Individual differences, such as age, sex, and condition, can greatly affect their contribution to population growth and consequently the same population size can lead to different population trajectories [15,16]. Understanding the evolutionary selection pressures operating in a species also requires understanding the demography [17].

It has been suggested that Plethodontid salamanders, which comprise about 70% of the living urodelan species, are a suitable group to use as an indicator of ecosystem health [18,19]. However, long-term studies on them are scarce [20] and demographic information for them is mainly based on life table and time series analyses of few terrestrial species from the USA and Europe [for a recent review, see [21]]. In general, these studies suggest that limiting factors, such as moisture, food and retreat or nesting sites may contribute to density-dependent population regulation [21]. Time series analysis on a population of the NW Italian *Speleomantes strinatii *showed that the population trajectory is stable and that the population was probably fluctuating near the environmental carrying capacity [22].

As most of the long-term population data on salamanders are from a small number of species, mainly North American *Plethodon *and *Desmognathus *species [e.g. [23,24]], and from a single species of the European genus *Speleomantes *[25,26], our understanding of the population regulation and demography of terrestrial salamanders is rather limited. Here we present an analysis based on a long-term data set on an underground population of *Speleomantes strinatii *using state-space modelling. This approach allows us to take into account both the inherent stochasticity in the demographic processes, and the uncertainty in the parameter estimates while using all of the available data to inform the model fit [27]. We explicitly model the underlying stochastic process, using Markov chain Monte Carlo (MCMC) simulation in a Bayesian framework [28-30].

## Results

Abundance estimates were obtained for each age class and sex group in all years (N = 13). Mean capture probabilities (CP) were relatively high ranging from 0.60 to 0.72, and there were no significant differences among groups (repeated measures ANOVA, F = 1.37, DF = 4, P = 0.335) or years (repeated measures ANOVA, F = 0.97, DF = 12, P = 0.492), as noted by Salvidio [31]. There was no relationship between mean snout vent length (SVL) and CP (Spearman's ρ = 0.10, n = 5, P = 0.87), but it may be noteworthy that the smallest age group (i.e. first year juveniles) had the lowest CP. Overall these results indicate that the abundance estimates were reliable and should be considered with relatively high confidence as CP ≥ 0.40 provide "...really good results in the typical removal study, having N of a few hundred and t = 3 to 6" [32:108, where t is the number of removal occasions].

**Figure 1:**
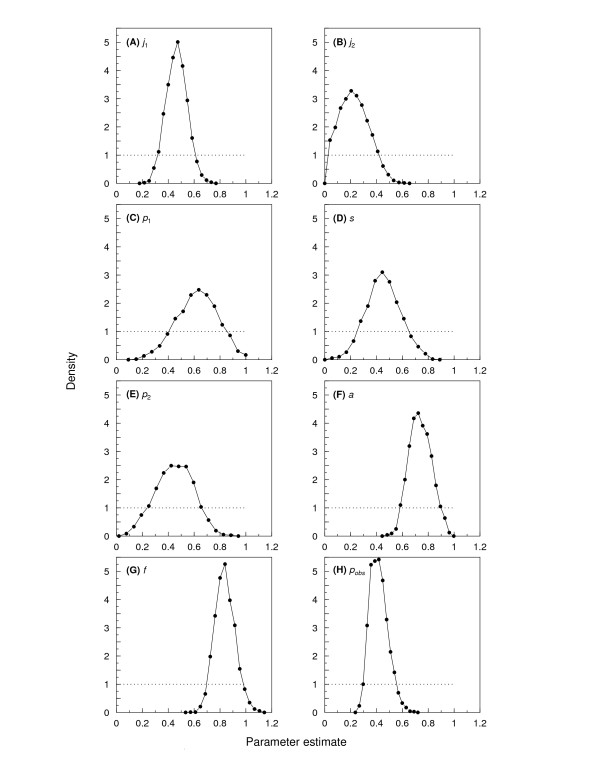
**Probability densities of the last 1,000 iterations of the WinBUGS estimation for the estimated demographic parameters**. (see Fig. 3 and Equation 2): *j*_1 _(A), *j*_2 _(B), *p*_1 _(C), *s *(D), *p*_2 _(E), *a *(F) and *f *(G). The medians, 2.5 and 97.5 percentiles are: *j*_1_: 0.45, 0.29, 0.61; *j*_2_: 0.20, 0.02, 0.44; *p*_1_: 0.60, 0.27, 0.89; *s*: 0.42, 0.17, 0.69; *p*_2_: 0.42, 0.14, 0.69; *a*: 0.72, 0.57, 0.90; *f*: 0.82, 0.68, 0.98, and *p*_obs_: 0.40, 0.29, 0.55. The priors are indicated by dotted lines.

The demographic parameter estimates are illustrated in Fig. 1. Two examples of different possible parameterisations of the Lefkovitch matrix are:(1)

corresponding to population growth rates, λ, of 0.97 and 0.98 respectively. The medians and 95% confidence intervals for the expected stable age-structure are 24% (20%,28%), 27% (22%,31%), and 49% (44%,54%) for juveniles, sub-adults and adults, respectively, reproductive values are 1.18 (0.93, 2.44) and 1.51 (0.92, 5.56) for sub-adults and adults relative to juveniles (whose reproductive value is scaled to 1), and the matrix elasticities (medians and 95% confidence intervals) are

[8]. These results indicate a stable population as the λ value was very close to 1 (median and 95% confidence interval: 0.95, 0.91, 0.99). At population equilibrium, we therefore expect half of the individuals among these three stage-classes to be reproductive adults. This agrees with the observed population structure, where the overall mean proportion of the modelled stage-classes (i.e. juveniles, subadults and adults) was 20%, 27% and 53%, respectively. The adult stage appears very important to population growth, having both the highest reproductive value (1.51) and the highest elasticity of all the matrix entries (0.42 for adult survival).

**Figure 2:**
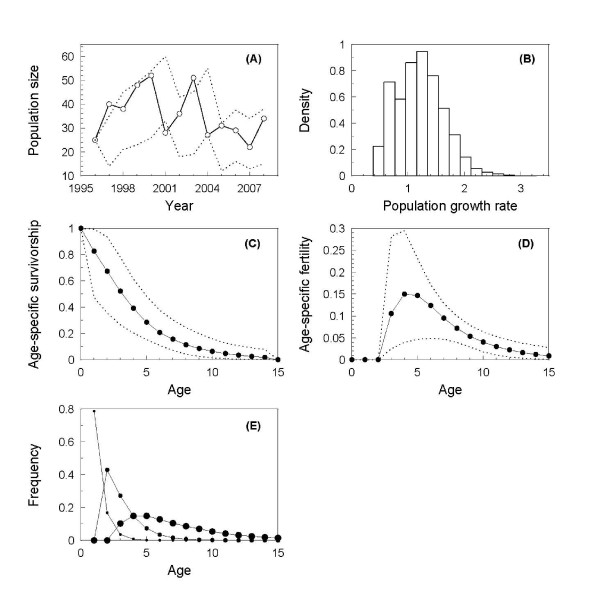
**Summary of the fitted demographic model**. (A) Observed female population size (open dots and solid line) and 95% confidence limit from the simulated population process (dotted line). (B) Frequency histogram of 1,000 rounds of the simulated population growth rate (calculated for each time step, i.e. 12 values for each simulation round). (C) Age-spefic survivorship and (D) fertility (medians denoted with solid line and filled dots, 95% confidence limit shown by dotted line) calculated from the transition matrix parameterised by sampling 1,000 combinations of parameter values from the last 5,000 posterior chains. (E) Age-within-stage distributions (the three stages are marked with small, medium and large dots for *J*_2_, *S *and *A*, respectively). These are scaled to proportions of individuals expected to be of a given age in each stage.

Fig. 1 shows the prior and posterior distributions of all the estimated population parameters, and Fig. 2A shows the observed female population sizes, and the 95% confidence intervals on the population sizes when the population renewal process was simulated using parameter estimates drawn from those posteriors (Equations 3-5). The resulting distribution of population growth rates is presented in Fig. 2B. Age-specific survival and fertility calculated as the expected values for a newborn individual are shown in Fig. 2C and 2D, and the age-within-stage distribution is shown in Fig. 2E.

## Discussion

Here we present the basic population demography of a population of the European plethodontid *Speleomantes strinatii *using a Bayesian state-space model built from long-term population data. Although not obtained through individual marking, abundance estimates of the different age and sex groups were obtained calculating specific capture probabilities, that, in the study population, were relatively high. Thus these basic demographic data can be considered reliable [32,33].

We found that the population growth rate estimate based on the parameterised Lefkovitch matrix model is very close to 1 giving us confidence that the further calculations based on this matrix assuming stable population are reliable [8]. This was further corroborated by the good match between the predicted and observed population stage-structure. The model also predicted the observed population reasonably well (Fig. 2B). We can therefore be reasonably confident that the other measures derived from the parameterised Lefkovitch model (age-specific survival and fertility and the age-within-stage distribution) are also reliable.

As this sub-population is part of a relatively dense population inhabiting a larger area, there is no reason to rule out immigration and emigration between years but without data on this we simply have to combine these processes with the survival and fecundity parameters. Better data on immigration and emigration would help to further narrow posterior distributions of all the parameter estimates. We also assumed 50:50 sex-ratio for births, and that the female fecundity is not limited by availability of mates. This is likely to be the case, as the adult population sex ratio (males/(males + females)) studied over 12 years was slightly male biased, being on average 0.57 (bootstrap 95% CI 0.53-0.65). This is probably due to the females entering the reproductive population one year later than males [25].

In terms of population growth, adult survival emerges as the most important determinant of population growth rate as indicated by the elasticity analysis. This finding is similar to an analysis on *Salamandra salamandra *(L) by Schmidt et al. [34]. This demographic feature may be common in salamander populations that possess high adult survival and lengthy reproduction, such as *Salamandra *and many large and medium-sized plethodontid species. In the case of terrestrial salamanders, such as *Speleomantes*, there is a considerable overlap in habitat and resource use by juveniles and adults, in contrast with the biphasic species that have aquatic larvae. This may enhance intraspecific density-dependent regulation in the terrestrial environment as suggested by Bruce [21]. However, the notion of the importance of adult survival to population growth based on this analysis has to be treated somewhat cautiously as the technique of basic elasticity analysis has been criticised for ignoring density dependence, stochasticity and correlations between the transition matrix entries [35,36]. Calculating the integrated elasticities [37,38] would be better than the more simplistic figures presented here, but we do not have annual estimates for all the demographic parameters in order to calculate correlations between the matrix entries and their variances as our approach needs all the data for estimating the parameters. In our case, the correlation estimates between matrix entries are based on different possible parameterisations of the demography (Table 1), not due to observed annual variation in those parameters. So, for instance the strong negative correlation between adult survival, *a*, and the transition probability from subadults to adults, *p*_2 _is probably more of an identifiability problem of the model [e.g. [39]] than reflecting the underlying biology of the species; it is possible to have a very similar fit to the data with a model with a relatively high *a *and low *p*_2 _as it is the other way round. Addressing this directly would require mark-recapture data where the individuals are followed over their whole lifespan. Unfortunately, this is not easily achieved with this species; newborns are less than 25 mm long including tail creating a major obstacle for marking with, for instance, elastomers (VIE) or pit tags which are suitable for adults. We would like to emphasise, however, that despite these shortcomings, we have used all the available data efficiently and parameterised a full demographic model for this species for the first time, simultaneously addressing the parameter uncertainty explicitly, and have therefore improved the current understanding of *Speleomantes *demography significantly.

**Table 1:** Correlation coefficients between the parameter estimates in the posterior chains.

Parameter	*j*_1_	*j*_2_	*p*_1_	*s*	*p*_2_	*a*	*f*	*p*_obs_
*j*_1_	1.00							

*j*_2_	-0.76	1.00						

*p*_1_	0.30	-0.43	1.00					

*s*	-0.35	0.32	-0.87	1.00				

*p*_2_	0.11	-0.08	0.43	-0.55	1.00			

*a*	-0.12	0.10	-0.44	0.48	-0.91	1.00		

*f*	-0.20	-0.01	-0.01	0.02	-0.02	-0.05	1.00	

*p*_*obs*_	-0.05	0.08	-0.15	0.16	-0.17	0.13	-0.13	1.00

## Conclusion

The modelling approach presented here is potentially very valuable in situations where following marked individuals over their whole lifetime is not feasible. For instance, the method introduced by Ricklefs [[40]; for use in Plethodontids, see [41])] calculate survival on the assumption that population growth rate is 1, an assumption we do not have to make. It was also recently shown [42] that state-space modelling approach produces results in close agreement with those based on mark-recapture techniques, highlighting further the potential of this approach.

## Methods

### Study species

*Speleomantes strinatii *is a medium-sized (total length < 115 mm) plethodontid salamander endemic to S France and NW Italy. It is found from the sea level up to about 2000 m a.s.l. on humid rocky outcrops, in the forest talus and in caves [43]. The aquatic larval stage is lacking and, during winter, females lay about 10 large terrestrial eggs that are attended for several months until hatching [43]. There are no field data on the proportion of successfully hatching embryos, and the only available data are from captive bred individuals. According to Durand [44], females lay 6-14 eggs but only half of them develop completely as females eliminate unfertilized or infected eggs from the clutch. In the case of a video surveilled female observed during the entire brooding period, only two out of nine attended eggs produced viable offsprings (Oneto et al. unpublished data). In this species, recruitment is seasonal and three immature body size groups (i.e. newborns, yearlings and subadults) may be recognised as separate age classes [45]. Males become sexually active at a snout-vent length (SVL) of about 50 mm when a mental gland, lacking in females and juveniles, becomes evident. A previous study based on dissections [25], demonstrated that females begin yolking at a SVL of 58 mm, and are probably reproductive at an older age than males.

From a conservation point of view, the species appears widespread and it is not considered globally endangered [43]. However, populations living in Mediterranean karstic areas that tend to concentrate inside caves or other artifical underground habitats, at least during unfavourable external environmental conditions, are highly exposed to disturbance, predation and or collection, and can thus be considered locally threatened.

### Data collection and field methods

The study site is an artificial cavity excavated during World War II near Busalla (province of Genova, NW Italy) and naturally colonised by salamanders [26]. Cretaceous marlstones constitute the geological substrate of the area and a true natural karstic system is lacking, thus artificial underground retreats constitute an extension of the natural superficial underground compartment available to salamanders. During dry summer periods salamanders concentrate inside these buffered habitats in which low temperatures and high moisture levels are present, while they are less active on the soil surface.

During July, from 1996 to 2008, the population abundance was estimated by a standardised temporary removal experiment [32], with three removal samples obtained every other day (i.e. the total sampling period was 96 hours). During sampling the population was considered demographically closed, because dispersion from and towards the underground cavity was prevented by high temperatures and dry weather conditions, typical of summer climate in the region.

Salamanders caught on the cavity walls were measured from the snout to the posterior end of the cloaca to the nearest mm (snout-vent length, SVL) and kept in ventilated plastic boxes inside the cavity. Sex was determined only in adults, as mature males possess a conspicuous mental gland that is absent in females and immatures of both sexes. In each year sample the population was well structured, as all age groups were present, indicating that there was no transient emigration, a phenomenon that may bias the estimates of population demographic parameters [46]. At the end of the experiment, all salamanders were released. To delimit body size components of each year sample correctly, the population structure was separated into first and second year juveniles, subadults and adults by means of FiSAT software [47]. This procedure gives more reliable results than using fixed SVL values, because the population structure shows year to year variations. Thus the number of different age class individuals was estimated separately with the generalized removal model M_bh _of CAPTURE software that allows for heterogeneity in capture probabilities [32]. For further details on the methods and the study population, see [22,31,48].

### Parameter estimation

**Figure 3:**
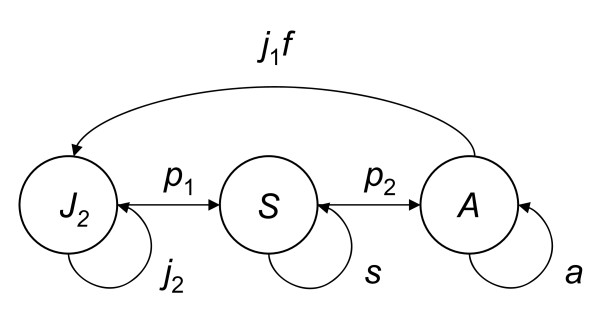
**Life cycle graph for *Speleomantes strinatii***. The stage-classes are *J*_2 _(juveniles born in the previous year), *S *(subadults) and *A *(adults), see text for further details.

We modelled the female population using a standard Lefkovitch model for population growth with birth-pulse dynamics and pre-breeding census [8]. The salamander life cycle (Fig. 3) corresponds to transition matrix **M**:(2)

where the parameters represent transitions between the different stages *J*_2 _(juvenile), *S *(subadults) and *A *(reproductive adults), from one year to the next (Fig. 3). Note that as we had data available for the annual number of juveniles born in the year of the census, we utilised this information to introduce a transitional stage *J*_1 _and wrote the fecundity contribution of the adults as *j*_1_*f*, where *f *is the expected number of female offspring produced by a female per year and *j*_1 _is the probability that this newly produced offspring survives until the age of one year, and therefore completes the transition from newly born to juvenile, *J*_2_. The parameters *p*_1_, *p*_2_, *j*_1_, *j*_2_, *s *and *a *were estimated in such a way that each year adults first reproduce (at rate *f*), then all individuals survive or die (with stage-dependent survival probabilities *p*_*j*1_, *p*_*j*2_, *p*_*s *_and *p*_*a*_), and finally individuals in stages *J*_1_, *J*_2 _and *S *will either continue to the next stage or stay where they are (with transition probabilities 1, γ_1 _and γ_2 _respectively - *i.e*. all new juveniles in *J*_1 _transition immediately to *J*_2 _before the end of the year). See Equation 4 for a mathematical description of this process.

We used a Bayesian framework for estimating the parameters of this demographic model, fitting the models using Monte Carlo Markov Chain (MCMC) techniques with the aid of the WinBUGS (version 1.4.3) statistical programming environment [28]. As very little prior knowledge on these demographic parameters is available, we used uninformative priors, using continuous uniform distributions as a starting point for each parameter. These were limited to 0 and 1 for all the other parameters except the fecundity parameter *f *for which the upper limit was set to 2 (as exploratory analyses indicated much smaller values for it). Note that this does not reflect the number of eggs laid, typically 6-14 in this species [44], but rather the number of hatched young per female. We also modelled the observation process explicitly, with a constant observation probability across all age classes and time periods [31], and hence the observed number in any class as binomially distributed from the true population at that time, with the probability of observation being a parameter of the model which is estimated from the data.

The WinBUGS model was run for 20,000 iterations with 5 chains allowing a "burn-in period" for 5,000 iterations. Convergence of the parameter estimates was confirmed with the Gelman-Rubin convergence statistic provided by the WinBUGS software, and in all cases the Monte Carlo error was less than 5% of the sample standard deviation (max. 2.2%). The observed autocorrelation in samples disappeared by setting the "thinning" to 50. In the analyses, we used the last 5,000 observations of the converged posterior chains. Since there was strong correlation between some of the model parameters (Table 1), we took 1,000 samples from these posteriors to parameterise the matrix model. All the subsequent simulations were run using MatLab 7.5.0 R2007b.

In addition to the estimation of the demographic parameters, we also simulated the population growth using the posterior distributions of the parameters (Table 1). Using the parameterised Lefkovitch matrix (Eq. 2) as a starting point, we then calculated the transition elasticities, stage-specific reproductive value (indicating the expected long-term contribution by each stage, given by the left eigenvector of the transition matrix), stable population structure, age-specific survivorship and fertility, and the age-within-stage distributions using the methods described in [8]. The population renewal process was simulated by taking all the variability associated with the estimated population parameters into account. This procedure was repeated with the 1,000 posterior samples, so that the population at round *i *would renew itself according to the following equations.(3)

Equation 3 represents the observation process in year t, where *J*_1_^*o*^, *J*_2_^*o*^, *S*^*o *^and *A*^*o *^are the observed numbers in the different stages, *J*_1 _*, *J*_2 _*, S* and A* are estimates of the true (unobserved) population sizes, *p*_obs _is the observation rate or capture probability and *nbin *is the negative binomial distribution formulation representing the number of unobserved individuals before *J*_1_^*o*^, *J*_2 _^*o*^, *S*^*o *^or *A*^*o *^individuals are observed with this capture rate; since we separate out the observation model from the underlying process model, this constitutes a state-space model [29]. Then the following three sets of equations govern the birth, death, and stage-transition of the individuals in year *t*:(4)

Working from top to bottom, the first equation governs the birth of new offspring into the transitional stage *J*_1_; this is used here in the MCMC model and informs estimates of the parameter *f*, but in the simulation it is used in year *t*-1, together with an observation process to assess the accuracy of observed juveniles in the following year (see below). In the middle four equations, *J*_1_', *J*_2_', *S*' and *A*' denote the number of individuals in stages *J*_1_, *J*_2_, *S *and *A *that survive that stage. In the final two equations, *J*_2_^+ ^and *S*^+ ^are the number of those survivors which move to the next stage (all survivors of stage *J*_1 _transition immediately to *J*_2_); *bin *and *pois *are the Binomial and Poisson distributions respectively. Finally:(5)

combines the calculation of estimated next generation stage sizes with the observation process to assess the accuracy of the simulation.

## Authors' contributions

JL and RR conducted the data analysis and modelling, and JL wrote the paper. SS collected field data, and both RR and SS contributed to writing. All authors read and approved the final manuscript.
